# mSphere of Influence: If virulence is energetically costly, how can it be maintained?

**DOI:** 10.1128/msphere.00312-23

**Published:** 2023-09-07

**Authors:** Kimberly Michele Davis

**Affiliations:** 1 W. Harry Feinstone Department of Molecular Microbiology and Immunology, Johns Hopkins Bloomberg School of Public Health, Baltimore, Maryland, USA; University of Kentucky College of Medicine, Lexington, Kentucky, USA

**Keywords:** bacterial cooperation, heterogeneity, population dynamics, virulence factors

## Abstract

Kimberly Davis works in the field of bacterial pathogenesis and studies heterogeneity in bacterial populations within host tissues. In this mSphere of Influence article, she reflects on how the paper “Stabilization of cooperative virulence by the expression of an avirulent phenotype” by M. Diard et al. (M. Diard et al., *Nature* 494:353–6, 2013, DOI: 10.1038/nature11913) impacted the way she thinks about bacterial population dynamics and the costs and benefits of producing virulence factors during infection.

## COMMENTARY

Virulence factors can be defined as components required to establish infection within a host. In the absence of a bacterial virulence factor, bacterial strains are impaired in establishing infection, and restoring expression of the factor rescues virulence (1). Expression of virulence factors can be energetically costly to a producing bacterial cell, while other cells can benefit from the expression of a virulence factor without the cost of producing it, a mechanism termed “cooperative virulence” (2). This can manifest as two phenotypes during infection: a virulence factor-expressing subpopulation with reduced fitness or slowed growth and a non-expressing subpopulation with more rapid growth kinetics (3, 4). Furthermore, genetic mutants can arise that completely lack the ability to express virulence factors but benefit from neighboring expressing cells, which are termed “cheaters.” These cells are avirulent and cannot go on to infect a subsequent host, but again, their growth is supported by other virulence factor-expressing members of the population (2). In Diard et al. (5), the authors explore these dynamics within populations of *Salmonella enterica* serovar Typhimurium (*S*. Typhimurium). Their research focuses on the expression of the critical virulence factor, the type-III secretion system-1 (*ttss-1*, T3SS-1, or T1), which was previously shown to be bistably expressed in culture and infection models, leading to two phenotypically distinct subpopulations (T1^+^ phenotypically virulent and T1^−^ phenotypically avirulent) (3, 4). They also examine the impact of genetically avirulent mutants, or cheaters, and determine how the abundance of each subpopulation impacts overall population size and cooperative virulence. The authors ask a key question in bacterial pathogenesis research: given the fitness costs associated with virulence gene expression, how does a strain maintain virulence? You would predict that virulence would be readily lost after infection is established, but successful pathogens clearly have evolved mechanisms to maintain virulence. Critically, the authors explore bacterial population dynamics in a mouse model of infection to assess the impact of fitness costs and selection in the context of host inflammation. They show the phenotypically avirulent T1^−^ population is vital to maintaining virulence (5); avirulent mutants arise more slowly with a larger T1^−^ population because these two avirulent populations replicate at similar rates. However, T1^−^ populations retain the ability to express virulence genes and can switch to T1^+^ as needed, thus maintaining virulence.

The authors initially studied subpopulation dynamics utilizing mathematical modeling-based approaches (5). A wild-type population of *S*. Typhimurium contains ~63% T1^−^ bacteria (4), which promotes stable infection of the small intestine in the mathematical model. If avirulent mutants are incorporated, they rapidly expand in numbers, resulting in population collapse. Reducing the proportion of T1^−^ cells speeds avirulent mutant takeover and population collapse since slow-growing T1^+^ cells are quickly outcompeted by the fast-growing avirulent mutants. These modeling-based predictions were then tested in a mouse model of infection, where bacterial genetic mutants were used to fine-tune the proportion of T1^+^ and T1^−^ subpopulations. They show that a heightened proportion of T1^−^ cells is critical to maintaining cooperative virulence by slowing the emergence of mutants. They confirm the avirulent mutants are cheaters, which benefit from the inflammation caused by T1^+^ cells without contributing to inflammation or incurring the fitness cost associated with it. They conclude that the presence of phenotypically avirulent cells is critical to maintaining population stability since these cells grow as rapidly as avirulent mutants but maintain the ability to express T3SS-1 as needed.

One major point of emphasis for this study was the fitness benefit of utilizing gene regulation to quickly, and reversibly, switch between phenotypes, compared to more irreversible genetic switches. Phenotypic switching typically occurs in specific subpopulations of bacteria, either prior to sensing a stimulus by a bet-hedging approach or in direct response to an environmental change, resulting in a division of labor (2, 6). These dynamics maintain the population as a whole, despite the fitness cost incurred by the subpopulation. The dynamics of this coordinated behavior over the course of infection were another point of emphasis, along with the importance of utilizing infection models to complement culture-based approaches to studying dynamics. While culture-based approaches defined differences in growth rates amongst the phenotypes (3, 6), it was necessary to move into an infection model to understand fitness costs and benefits in the presence of host inflammation. In the years since this paper was published, it has become clear that these findings extend well beyond *Salmonella* and are broadly applicable to populations of bacterial pathogens. For me, this study emphasized the need to develop single-cell approaches to characterize heterogeneity and dynamics within host tissues. In my own lab, we study phenotypic heterogeneity and cooperative virulence in *Yersinia pseudotuberculosis* populations within mouse models of infection (7, 8) and recently began to focus on the link between virulence factor expression, fitness costs, and reduced antibiotic susceptibility (9, 10). We developed fluorescence-based strategies to simultaneously detect growth rate heterogeneity during infection and approximate intracellular concentrations of antibiotic, which we are utilizing to understand the mechanisms underlying bacterial survival during antibiotic treatment (10 – 13). Fluorescent reporters allow us to visualize the spatial location and abundance of subpopulations within host tissues, and we are currently optimizing isolation approaches for downstream analyses.

Heterogeneity is critical to study due, in large part, to the implications for antimicrobial susceptibility and effective treatment strategies. In subsequent studies, the authors showed that T1^+^ cells invade gut tissues, remain viable, and preferentially survive antibiotic treatment during infection (14). In a culture-based study, they showed T1^+^ cells preferentially survive antibiotic treatment due to slowed growth (6). These studies emphasize a linkage between virulence factor expression, slowed growth, and the impact on antibiotic susceptibility and suggest antibiotic treatment can select for resistance and also virulence when virulence factor expression comes at a fitness cost. This is disheartening for antibiotic treatments, but it may suggest we could improve treatment efficacy by modulating the host response to promote more efficient bacterial clearance. Diard et al. suggest another possible solution: modulating the bacterial population by adding genetically avirulent cells to promote the collapse of the virulent population. With many research groups working to tackle these issues, it will be very exciting to see the solutions we discover in the coming years.
